# Alcohol and brain structure across the lifespan: A systematic review of large‐scale neuroimaging studies

**DOI:** 10.1111/adb.13439

**Published:** 2024-09-24

**Authors:** Hollis C. Karoly, Katelyn T. Kirk‐Provencher, Joseph P. Schacht, Joshua L. Gowin

**Affiliations:** ^1^ Department of Psychology Colorado State University Fort Collins Colorado USA; ^2^ Department of Radiology, School of Medicine University of Colorado Anschutz Medical Campus Aurora Colorado USA; ^3^ Department of Psychiatry, School of Medicine University of Colorado Anschutz Medical Campus Aurora Colorado USA

**Keywords:** alcohol consumption, alcohol exposure, brain structure, MRI, neuroimaging

## Abstract

Alcohol exposure affects brain structure, but the extent to which its effects differ across development remains unclear. Several countries are considering changes to recommended guidelines for alcohol consumption, so high‐quality evidence is needed. Many studies have been conducted among small samples, but recent efforts have been made to acquire large samples to characterize alcohol's effects on the brain on a population level. Several large‐scale consortia have acquired such samples, but this evidence has not been synthesized across the lifespan. We conducted a systematic review of large‐scale neuroimaging studies examining effects of alcohol exposure on brain structure at multiple developmental stages. We included studies with an alcohol‐exposed sample of at least *N* = 100 from the following consortia: ABCD, ENIGMA, NCANDA, IMAGEN, Framingham Offspring Study, HCP and UK BioBank. Twenty‐seven studies were included, examining prenatal (*N* = 1), adolescent (*N* = 9), low‐to‐moderate‐level adult (*N* = 11) and heavy adult (*N* = 7) exposure. Prenatal exposure was associated with greater brain volume at ages 9–10, but contemporaneous alcohol consumption during adolescence and adulthood was associated with smaller volume/thickness. Both low‐to‐moderate consumption and heavy consumption were characterized by smaller volume and thickness in frontal, temporal and parietal regions, and reductions in insula, cingulate and subcortical structures. Adolescent consumption had similar effects, with less consistent evidence for smaller cingulate, insula and subcortical volume. In sum, prenatal exposure was associated with larger volume, while adolescent and adult alcohol exposure was associated with smaller volume and thickness, suggesting that regional patterns of effects of alcohol are similar in adolescence and adulthood.

## Abstract 2

This systematic review of large‐scale neuroimaging studies examining the effects of alcohol exposure on brain structure across multiple developmental stages showed that adult alcohol consumption was characterized by smaller volume and thickness in frontal, temporal, and parietal regions, and reductions in insula, cingulate, and subcortical structures. Adolescent alcohol consumption had similar effects, but with less consistent evidence for smaller cingulate, insula, and subcortical volume. This suggests that regional patterns of effects of alcohol are similar in adolescence and adulthood.

## 1 INTRODUCTION

Heavy alcohol consumption is a leading cause of death and disease worldwide, leading to increased rates of cancer, liver disease and risk for accidents.^1^, ^2^ Alcohol exposure can begin as early as conception, and alcohol consumption is common throughout the lifespan; in the United States, 86% and 82% of men and women, respectively, report alcohol use at some point in their lifetimes.^3^ The mean age of initiation of alcohol use is 17,^4^ when the brain is still developing. A typical developmental course of brain structure involves increases in grey matter volume and density throughout the brain before puberty, followed by a rapid decline during and after puberty.^5^​ Grey matter volume loss continues more slowly during middle age before accelerating in older adults.^6^​​ Alcohol is thought to affect brain structure primarily through directly causing neuronal death. Neuronal death related to alcohol use can be caused by acetaldehyde exposure,^7^ oxidative stress,^8^ decreases in glucose metabolism following acute alcohol intake,^9^ and liver damage, as toxins that are normally processed and removed by the liver can build up and enter the brain.^10^ Heavy alcohol exposure can also reduce the number of stem cells that help to generate new neurons.^8^ Despite evidence that alcohol exposure at any time can affect brain structure, it remains unclear to what extent the age at which individuals consume alcohol determines its effects on brain structure.

The most common method to study alcohol's effect on brain structure is magnetic resonance imaging (MRI), and recent evidence suggests that large sample sizes are necessary to provide reliable estimates of relationships between brain structure and function.^11^ Given the recent attention to guidelines for healthy alcohol consumption, and efforts by some countries to modify guidance,^12^ it is important to have high quality evidence about the effects of alcohol on the brain across developmental stages. Many previous studies examining alcohol's effects on brain structure have included modest samples sizes (e.g., <50 participants per group). While these smaller studies provide useful cohort‐specific estimates of the effects of alcohol on the brain, a number of recent efforts have been made to acquire very large sample sizes to better characterize alcohol's effects on the brain on a population level, including multi‐site studies and consortia that combine samples from many laboratories into a single repository.^13^, ^14^, ^15^, ^16^, ^17^, ^18^, ^19^ These efforts often examine different development periods, such as adolescence, young adulthood, or middle and later adulthood. However, to date, no systematic review has synthesized these large‐scale studies to describe how alcohol affects brain structure across the lifespan.

This review leverages data from large‐scale consortium studies to examine the effects of alcohol exposure and consumption on brain volume across developmental periods. Specifically, we examined prenatal alcohol exposure and alcohol use in adolescence and adulthood, with adult studies divided by amount of alcohol consumption (i.e., low‐to‐moderate exposure and heavy exposure). To ensure that studies were sufficiently powered to detect brain‐wide associations^11^ and to covary for important potential confounding factors such as age, sex, health history, socioeconomic status and other substance use, only studies that included samples of at least 100 alcohol‐exposed participants from the Framingham Offspring Study, UK Biobank, the National Consortium on Alcohol and Neurodevelopment in Adolescence (NCANDA), the Adolescent Brain Cognitive Development (ABCD) Study, the Enhancing Neuro Imaging Genetics Through Meta‐Analysis (ENIGMA) Project, the Imaging and Genetic (IMAGEN) Project and the Human Connectome Project (HCP) were included (see Table S1 for a description of each consortium). We hypothesized that alcohol exposure would be associated with similar patterns of smaller brain volumes (relative to controls) across all developmental stages. We also evaluated whether the effects of alcohol differed by sex.

## 2 MATERIALS AND METHODS

This review follows the Preferred Reporting Items for Systematic Reviews and Meta‐Analyses (PRISMA) guidelines.^20^ The protocol was pre‐registered through the Open Science Framework (OSF, protocol can be accessed at https://osf.io/hfsu3, currently embargoed) on 1 August 2023. The last search date took place 19 June 2023.

### 2.1 Search strategy

Electronic literature searches were conducted through MEDLINE, PubMed, Web of Science and Embase on 16–19 June 2023. The full electronic search strategies for MEDLINE, PubMed, Web of Science and Embase are included in the supporting information. For brevity, in Table 1, we present the Embase search (which includes MEDLINE). Note that the search strategy covered four separate ‘concepts’: (1) alcohol‐related terms, (2) brain‐related terms, (3) neuroimaging‐related terms and (4) the names or grant numbers of the seven consortia included in the review. The final search combined 1 AND 2 AND 3 AND 4.

**TABLE 1:** Search details: Broad concepts and search terms (Embase).

Broad concept	Specific search terms
1. Alcohol	‘alcoholic intoxication’/exp OR ‘alcoholism’/exp OR ‘alcohol drinking’/exp OR ‘ethanol’/exp OR (alcohol* OR aud OR ‘heavy drinking’ OR ‘binge drinking’ OR ‘teen drinking’ OR ‘teenage drinking’ OR ‘adolescent drinking’ OR ‘youth drinking’ OR intoxicat* OR ethanol OR beer OR wine)
2. Brain	‘brain’/exp OR (brain OR cereb* OR ‘gray matter’ OR ‘surface area’ OR cortic* OR cortex)
3. Neuroimaging	‘neuroimaging’/exp OR ‘magnetic resonance imaging’/exp OR (‘magnetic resonance imaging’ OR mri OR neuroimag*)
4. Consortium Name or Grant Number	‘adolescent brain cognitive development study’ OR ABCD OR ‘U24 DA041147’ OR U24DA041147 OR ‘enhancing neuro imaging genetics through meta‐analysis’ OR ENIGMA OR ‘U54 EB020403’ OR U54EB020403 OR ‘Framingham Offspring Study’ OR fos OR ‘N01 HC25195’ OR N01HC25195 OR ‘Human Connectome Project’ OR hcp OR U54MH091657 OR U01AG052564 OR ‘U54 MH091657’ OR ‘U01 MH109589’ OR ‘U01 AG052564’ OR ‘imagen study’ OR IMAGEN OR ‘MRF_MRF 0580009 RG DESR C0759’ OR ‘national consortium on alcohol and neurodevelopment in adolescence’ OR NCANDA OR ‘U24 AA021695’ OR ‘UK Biobank’ OR MC_QA137853 OR MC_PC_17228’
Search combined 1 AND 2 AND 3 AND 4	

### 2.2 Eligibility criteria and article screening process

Results of the search procedure described above were uploaded into Covidence, which automatically removed duplicates and generated 404 distinct articles to screen. Covidence was used for management of all steps of the review/screening and data extraction process. First, title and abstract screening was conducted for the 404 articles. Two reviewers independently reviewed each title and abstract, and conflicts were resolved via full group consensus from all authors. Included articles were required to be (1) peer‐reviewed, (2) available in English, (3) empirical studies that analysed alcohol use/exposure in relation to brain structural changes and (4) to have used MRI to assess brain structure (i.e., volume, thickness and area). Reviews, reports, meta‐analyses, dissertations, theses, book chapters, abstracts, case studies, guidelines, expert opinions, commentaries and other non‐peer reviewed work were excluded.

More detail on inclusion/exclusion criteria applied are listed in Table 2. Regarding the inclusion criterion that required articles to have an alcohol‐exposed sample of at least *n* = 100, meta‐analyses comparing brain volume between heavy drinkers and light drinkers suggest the greatest differences between these groups in regional brain volume represent large effect sizes (i.e., Cohen's *d >* 1.0).^21^, ^22^ Assuming the average difference in brain volume between these groups represents a medium effect size (*d* = 0.5), a group sample size of 105 would provide 95% power to detect such an effect. Thus, a sample size of *n* > 100 per group should provide adequate power to detect effects of alcohol in brain regions with clinically meaningful effects^23^ and allow for adjustment of covariates that could confound results, such as age, sex, socioeconomic status and co‐occurring substance use.

**TABLE 2:** Detailed PICOS criteria.

Component	Inclusion criteria	Exclusion criteria
**Population**	• Human data from one of these 7 neuroimaging consortia: ABCD, ENIGMA, Framingham Offspring Study, the Human Connectome Project, IMAGEN, NCANDA, UK Biobank • Alcohol exposed sample of at least *N* = 100	• Data not from one of the 7 consortia • Alcohol‐exposed sample below *N* = 100
**Intervention/Determinant**	Alcohol exposure	No low‐alcohol‐exposure group or no low‐using individuals in the sample
**Comparison**	Low‐alcohol exposure group or non‐drinking group	The comparison group was polysubstance use only Explored family history/genetic risk as a predictor of brain structure but did not test effects of alcohol use/exposure.
**Outcome**	• Brain structural MRI measure (volume, thickness, structural changes)	• Examined the relationship between alcohol and brain structure “backwards” (i.e., used measures of brain structure to predict future drinking) • Did not assess association between alcohol use/exposure and brain structure • Used neuroimaging technique other than structural MRI
**Study Design**	• Compared high alcohol use/exposure group vs. a no/low alcohol use/exposure group (cross‐sectional/case–control or cohort design) OR • Alcohol use as a continuous predictor in a regression model, where alcohol use in the sample ranges from low use to higher use (cross‐sectional or cohort design) OR • Explore longitudinal changes in brain volume as a function of changes in drinking over time (longitudinal or cohort design)	• Meta‐analysis • Systematic Review

Fifty‐eight articles passed title and abstract screening and underwent full‐text review. Two reviewers independently reviewed each text, and conflicts were resolved by an independent third reviewer. Twenty‐seven articles passed full‐text screening and underwent data extraction for inclusion in this review.

### 2.3 Data extraction

**FIGURE 1:**
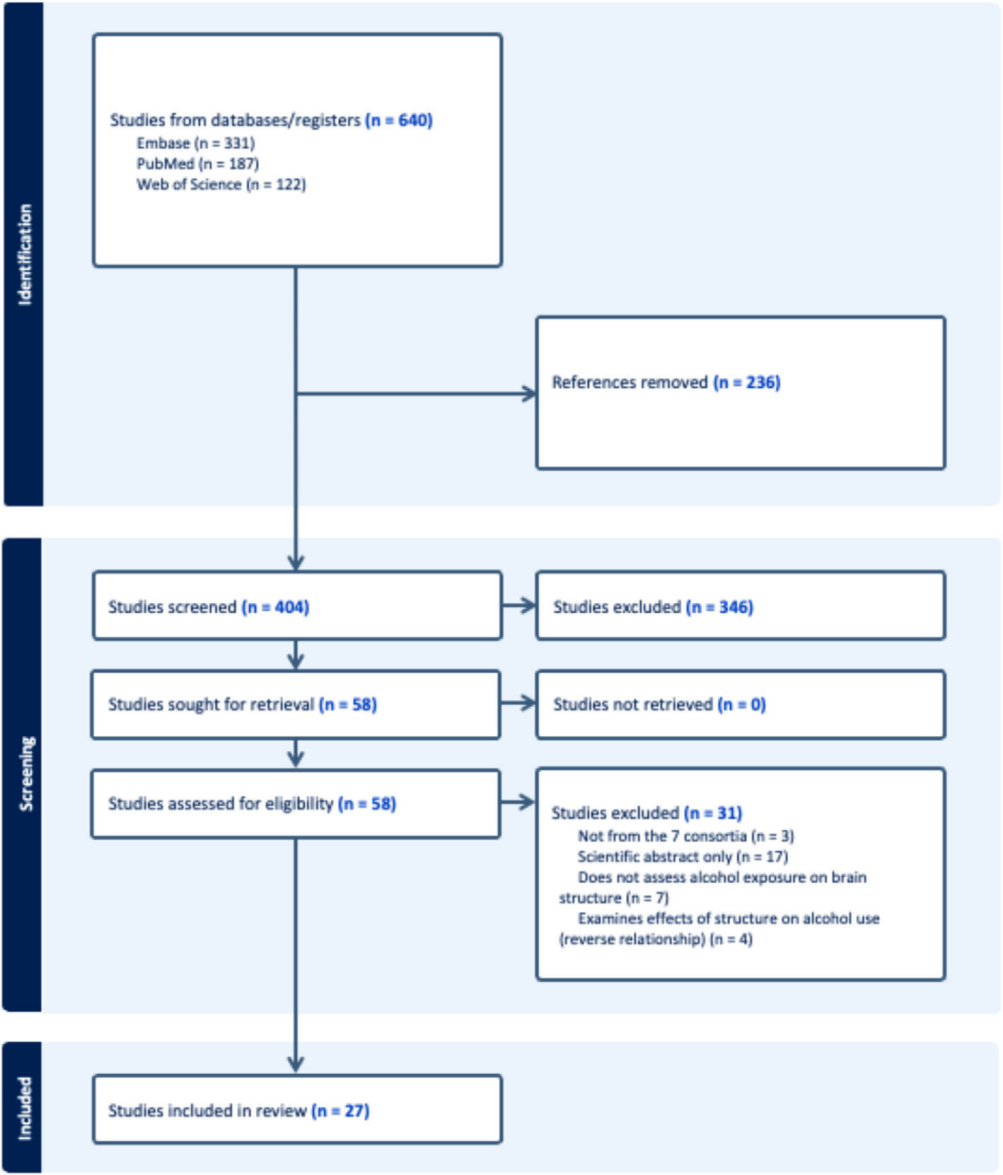
Prisma flow chart depicting the flow of identification and screening of studies for inclusion in this review.

Two extractors independently extracted the data from each included article using a template the four authors created collaboratively in Covidence. Discrepancies in the extractions were resolved by an independent third reviewer. Information extracted for each article was multi‐site study or consortium, study design, participant characteristics (alcohol exposure, inclusion/exclusion criteria, total sample, total alcohol‐exposure sample, age range of sample, sex, and race), whether the study reported brain structure differences by sex, results (i.e., was alcohol associated with smaller/larger/no difference in brain volume, and in what areas) and which covariates were included. The main covariates extracted included socioeconomic status, sex, age, race, ethnicity, other substance use, other major psychiatric diagnosis and other medical condition. Results are reported and synthesized in narrative form. A visual depiction of the article selection and data extraction process is shown in Figure 1 (PRISMA flowchart), and information about each study that was included is listed in Table 3.

**TABLE 3:** Large‐scale neuroimaging studies that met criteria for this review.

Study	Consortium	Total sample	Report brain differences by sex	Included other substance use as a covariate	Alcohol use comparison	Age range or mean age reported	Correction for multiple comparisons	ROB/Quality of study
**Prenatal exposure**								
Lees 2020	ABCD	9719	No	Yes	Prenatal alcohol exposure as a dichotomous variable; also tested associations between total drinks consumed during pregnancy and brain structure	9.0–10.9	False discovery rate	Good
**Adolescent exposure**								
Infante et al.^29^	NCANDA	242	No	No	Regression models in which the predictor is binge vs. non‐binge; regression models in which predictor is number of binge drinking episodes in past year	12–21	Bonferroni correction	Good
Luo et al.^28^	NCANDA	724	Yes	Yes	Drinking vs. no drinking in past year; number of drinking days in past year	Mean 16.02, SD = 2.45	Bonferroni correction	Good
Ottino‐Gonzále et al.^25^, ^a^	IMAGEN	297 hazardous drinking adolescents; 594 controls	No	No	Hazardous drinkers vs. controls	14–19	False discovery rate	Good
Phillips et al.^30^	NCANDA	803	No	Yes	Alcohol use: no/low, moderate, heavy and heavy binge	12–21 at baseline	False discovery rate	Good
Pfefferbaum et al.^33^	NCANDA	808	Yes	No	No/low vs. moderate to high alcohol consumption; also looked at associations with number of binges per year and associations with lifetime drinks in the moderate to high alcohol consumption group	12–21.9	None reported	Good
Pfefferbaum et al.^32^	NCANDA	483	No	No	Moderate alcohol use vs. no/low use and heavy use vs. no/low use	12–21 at entry	*p* ≤ 0.01	Good
Robert et al.^35^	IMAGEN	726	Yes	No	Drunkenness frequency and alcohol drinking units	12.9–23	Bonferroni correction	Good
Sullivan et al.^31^	NCANDA	548	Yes	No	No/low vs. moderate‐heavy drinking	12–21 at baseline	Bonferroni correction	Good
Sun et al.^34^	NCANDA	657	Yes	No	No/low vs. moderate/heavy alcohol exposure	12–22 at baseline	False discovery rate	Good
**Low to moderate adult exposure**								
Daviet et al.^39^	UK Biobank	36 678	Yes	Yes	Abstainers; <1 unit/day; 1–2 units/day; 2–3 units/day; 3–4 units/day; at least 4 units/day.	Approx. 45–80	*P* < 1.64 × 10–4 based on Holm Method	Good
Evangelou et al.^46^	UK Biobank	10 143 (included T1‐weighted sample only)	No	Yes	Alcohol consumption varying across a range of drinking levels	40–69	*p* < 0.017	Good
Hedges et al.^44^	UK Biobank	18 278	No	Yes	Regression model with drinking frequency levels (Daily/almost daily; 3–4 times/week; once or twice/week; 1–2 times/month; special occasions; never) as predictors	44–80	Not applicable (small number of comparisons)	Good
Matloff et al.^42^	UK Biobank	6213	No	Yes	No alcohol use, 1–6 g/day, 6–12 g/day, 12–24 g/day, 24–48 g/day, over 48 g/day	45–80	Not applicable (small number of comparisons)	Good
Morris et al.^37^	HCP	706	Yes	Yes	Total drinks in past 7 days and heavy episodic drinking past 12 months (Frequency of drinking 5+ drinks in past 12 months; never; 1–11 days/year; 13 days/month; weekly or greater	22–37	False discovery rate	Good
Ning et al.^43^	UK Biobank	17 308 (training + test sample); training sample = 5193, test sample = 12115	No	Yes	Abstainers, special occasions only, 1–3 times per month, 1–2 times per week, 3–4 times per week, daily or almost daily	45–80	Not applicable (small number of comparisons)	Good
Paul et al.^36^	Framingham Offspring	1839	Yes	No	Abstainers, former drinkers, low (1–7 drinks/wk.), moderate (8–14 drinks/wk.), high (greater than 14 drinks/wk.)	33 to 88	Not applicable (small number of comparisons)	Good
Topiwala et al.^45^	UK Biobank	25 378	Yes	No	Never drinker, previous drinker, <7 units/week, 7–12 units. 12–8 units. 18–28 units. Over 28 units	40–69 years at recruitment	False discovery rate	Good
Zhao et al.^40^	UK Biobank	8137	No	Yes	Abstainers (<1 unit/week), 1 ≤ 7 units/week, 7 ≤ 14 units/week, 14 ≤ 21 units/week, 21 ≤ 30 units/week, and >30 units/week	45–79	False discovery rate	Good
Zhao et al.^38^	HCP	293	No	No	Age at first drink less or greater than 21, past year binge drinking versus non binge drinker	22‐36F	False discovery rate	Fair
Zhao et al.^41^	UK Biobank	24 784 for cross sectional; 3070 of those include in longitudinal follow‐up	Yes	Yes	Cross sectional analysis: alcohol use scores based on AUDIT, longitudinal analysis: alcohol intake frequency: daily drinking; 3–4times/week; 1–2 times/week; 1–3 times/month; special occasion; never	45–81	Bonferroni correction	Good
**Heavy adult exposure**								
Chye et al.^51^	ENIGMA	3805	No	No	Alcohol dependent vs. nondependent individuals	Mean 32.9, SD 11.3	False discovery rate	Good
Grace et al.^50^	ENIGMA	966	Yes	Yes	Alcohol dependent vs. non‐dependent; monthly standard drinking dose	AD males = 34.21, AD females 32.68, control males = 30.34, control females = 29.48	False discovery rate	Good
Li et al.^21^	HCP	436	Yes	No	Binge drinkers (4 or more drinks for women and five or more drinks for men in single day) vs. non‐binge drinkers (grouped men and women separately)	22–36	Bonferroni correction	Good
Mackey et al.^49^	ENIGMA	3240	No	No	Alcohol dependence vs no alcohol dependence	~18–50	False discovery rate	Good
Navarri et al.^47^	ENIGMA	1245	No	No	Alcohol dependent cases vs non‐ dependent controls	12–60 (total ENIGMA sample)	False discovery rate	Good
Ottino‐González et al.^25^ ^a^	ENIGMA	745 adults with AD and 979 non‐dependent controls	No	No	Alcohol dependent vs non‐dependent controls	18–56	False discovery rate	Good
Rossetti et al.^48^	ENIGMA	986	Yes	Yes	Alcohol dependent cases vs controls; within alcohol dependent tested association with monthly standard drinks	18–68	False discovery rate	Good

Abbreviation: ROB, risk of bias.

^a^ Study that had data for multiple categories, and thus, this study was included under multiple sections of this table.

### 2.4 Quality assessment

The NIH Quality Assessment Tool for Observational Cohort and Cross‐Sectional Studies was used. Articles were rated on 14 items. The 14 items are listed for reference in the supporting information. Items cover quality domains such as statistical rigour, study design and transparency of methods. Following recommended guidance for this tool, 1 point was assigned for each ‘Yes’ answer and 0 points were assigned for each ‘No’, ‘Cannot Determine’ or ‘Not Reported’ answer. If an item was categorized as N/A, this was not counted against the total score. Scoring was conducted by calculating the total score (i.e., number of ‘Yes’ answers) divided by either 14 or, if any items were rated as N/A, that item was subtracted from the denominator. For example, if two items were rated N/A for a given article, the denominator for that article was 12. Based on these calculations, articles could be rated as ‘Poor’ (≤34% ‘Yes’), ‘Fair’ (35–63.9%) or ‘Good’ (≥64%). The final scoring system was adapted from a scoring system used previously.^24^

## 3 RESULTS

Our search revealed 640 references matching our criteria across the three databases. After removing duplicates, 404 studies were left for screening. After screening abstracts, 346 studies were excluded, leaving 58 for full‐text review. At this stage, 31 studies were excluded, leaving 27 for extraction, which examined prenatal (*n* = 1), adolescent (*n* = 9), low‐to‐moderate adult (*n* = 11) and heavy adult (*n* = 7) alcohol exposure. Note that this total is 28, as one study^25^ reports on adolescent data from IMAGEN as well as adult (heavy exposure) data from ENIGMA.

Due to substantial heterogeneity in analysis, age of samples and reporting, meta‐analysis was inappropriate, and this review will focus on identifying common and distinct effects of alcohol exposure and brain volume, thickness and surface area across developmental stages, grouped by prenatal, adolescent and adult exposure. For studies of volume, analyses typically corrected for intracranial volume and other potential confounds. Given the wide spectrum of alcohol exposures for adults, we divided this phase into studies examining low and moderate exposure versus those examining heavy exposure. Each consortium had its own specific drinking definitions (see Table S1), which corresponded with our 4 broad categories (prenatal, adolescent exposure, low‐moderate adult exposure and heavy adult exposure). In general, the adult heavy exposure studies primarily considered samples with alcohol use disorder (AUD) or binge drinking (defined as four or more drinks for women and five or more drinks for men in a single day). The adult low‐to‐moderate exposure studies considered a broader range of drinking patterns (e.g., ranging from monthly to weekly drinking, and considering up to approximately 7–8 drinks per week as light drinking, and approximately 14–15 drinks per week in the moderate range; see Table S1). The adolescent studies (from IMAGEN and NCANDA) defined drinking either by AUDIT scores (hazardous drinking defined by an AUDIT score or 8 or greater vs. non‐hazardous drinking), or used modified Cahalan criteria,^26^ which take into account both frequency and consumption (see Table S1). The summary of findings is presented in Table 4, and greater detail is provided in Table S2.

**TABLE 4:** Group comparisons of brain regions affected across the lifespan due to alcohol exposure.

Brain structure	Alcohol exposure timeframe			
	Prenatal exposure: children & adolescents^a^	Adolescence & early adulthood: initiation and use^b^	Mid‐ to older‐ adulthood: light to moderate use^b^	Mid‐ to older‐ adulthood: chronic use^b^
Global measurements				
Whole brain				
Total cerebral volume	↑^27^		↓^36^, ^39^, ^43^, ^46^	↓^48^
Grey matter				
Total volume/thickness	↑ volume^27^ ≈ thickness^27^	↓ volume^32^, ^33^ ↓ thickness^25^	↓ volume^39^, ^45^, ^46^ ≈ volume^38^ ↓ thickness^38^	↓ volume^48^, ^50^ ↓ thickness^25^
Frontal lobe				
Total frontal		↓ volume^32^, ^33^	↓ volume^39^, ^45^	
Total prefrontal cortex		↓ volume^35^	↓ thickness^37^	
Frontal gyri (*superior, middle, inferior, medial frontal; orbitofrontal; frontal pole*)	↑ area^27^ ↑ volume^27^	↓ thickness^34^ ≈ thickness^34^ ↓ volume^28^, ^29^, ^32^	↓ thickness^37^ ↓ volume^40^, ^46^ ^c^	≈ volume^48^ ↓ thickness^47^, ^49^ ≈ thickness^47^
Precentral/paracentral lobule/gyrus	↑ area^27^ ↑ volume^27^	↓ volume^29^	↓ thickness^37^ ↓ volume^40^, ^45^ ^c^	↓ thickness ≈ thickness^47^
Parietal lobe				
Total parietal		↓ thickness^33^ ≈ volume^32^	↓ volume^39^, ^45^	
Superior/inferior parietal lobe; precuneus	↑ area^27^ ↑ volume^27^	≈ thickness^34^ ↓ volume^29^	↓ thickness^42^ ↓ volume^40^ ^c^	↓ thickness^49^ ≈ thickness^47^
Postcentral, supramarginal gyri	↑ area^27^ ↑ volume^27^ ↑ thickness^27^	≈ thickness^34^ ↓ volume^28^, ^29^	↓ thickness^37^ ↓ volume^45^	↓ thickness^49^ ≈ thickness^47^
Temporal lobe				
Total temporal		↑, ↓ volume^33^ ^d^ ↓ volume^35^ ≈ volume^32^	↓ volume^39^, ^45^	
Entorhinal cortex		≈ volume^29^		≈ thickness^47^
Temporal gyri (*superior, middle, inferior, transverse temporal; parahippocampal; temporal pole*)	↑ area^27^ ↑ volume^27^ ↑ thickness^27^	≈ thickness^34^ ↓ volume^28^, ^29^ ≈ volume^29^	↓ thickness^37^, ^42^ ↓ volume^40^ ^c^	↓ volume^52^ ↓ thickness^47^, ^49^ ≈ thickness^47^
Superior temporal sulcus	↑ area^27^ ↑ volume^27^	↓ volume^29^		≈ thickness^47^
Occipital lobe				
Total occipital		≈ volume^32^, ^33^	↓ volume^40^, ^45^ ^c^	
Occipital gyri (*fusiform, lateral occipital, lingual*); cuneus; calcarine sulcus	↑ area^27^ ↑ volume^27^ ↑ thickness^27^	≈ thickness^34^ ↓ volume^2^, ^4^	↓ thickness^37^ ↓ volume^45^	↓ thickness^47^, ^49^ ≈ thickness^47^
Subcortical and limbic structures				
Cingulate				
Total cingulate		↓ thickness^33^ ↓ volume^32^	↓ volume^46^ ↓ volume^39^	
Anterior, middle, posterior cingulate; isthmus; paracingulate gyrus	↑ area^27^ ↑ volume^27^	≈ thickness^29^ ↓, ≈ volume^29^	↓ thickness^37^, ^42^ ↓ volume^40^, ^41^ ^c^	↓ volume^52^ ↓ thickness^47^, ^49^
Hippocampus	↑ volume^27^	↑ volume^30^ ^e^ ↓ volume^30^	↓ volume^42^, ^44^	↓ volume^47^, ^48^, ^49^, ^50^, ^51^
Insula		≈ thickness^34^ ↑ thickness^33^ ↓ volume^29^ ≈ volume^32^	↓ thickness^37^ ↓ volume^39^, ^46^	↓ volume^52^ ↓ thickness^49^ ≈ thickness^47^
Amygdala	↑ volume^27^	↑ volume^30^ ↓ volume^30^	≈ volume^45^ ↓ volume^39^, ^41^	↓ volume^47^, ^48^, ^49^, ^50^, ^51^, ^52^
Striatum				
Caudate, putamen			↓ volume^39^	↓ volume^47^, ^48^, ^49^, ^51^
Nucleus accumbens			↓ volume^41^	≈ volume^48^ ↓ volum^47^, ^49^, ^51^
Pallidum			↑ volume^39^	≈ volume^47^, ^51^ ↓ volume^48^
Thalamus, ventral diencephalon	↑ volume^27^		↓ volume^45^, ^46^	↓ volume^47^, ^48^, ^49^, ^51^
Cerebellum				
Total cerebellum	↑ volume^27^	↓ volume^31^	↓ volume^45^	↑ volume^48^, ^52^ ^f^ ↓ volume^48^ ^f^
Brain stem				
Pons			↓ volume^39^, ^45^	

*Note*: References for each finding are indicated by superscript numerical citations. ↑ and ↓ indicate alcohol exposure was associated with greater or smaller volume/thickness/surface area, respectively, ≈ indicates the reported absence of a significant effect of alcohol exposure, and empty cells indicate that effects or lack of effects were not reported in the studies included in this review. Adolescent studies were longitudinal. All other studies reported were cross‐sectional.

^a^ Reported results indicate comparisons between children or adolescents with prenatal alcohol exposure relative to those without prenatal exposure.

^b^ Reported results indicate comparisons between individuals (i.e., adolescents, young adults, or older adults) who drink alcohol (i.e., initiation of alcohol use, light, moderate, or chronic alcohol use) relative to individuals who do not drink alcohol/drink low amounts.

^c^ Zhao et al.^40^ reported widespread smaller volumes associated with greater alcohol consumption, but they applied a stringent correction for multiple comparisons (*q* < 1.72E−4), and these regions did not survive correction. For completeness, we report the regions they list as showing a relationship, given that their correction is more conservative than the typical paper in this literature.

^d^ Pfefferbaum et al.^33^ reported that participants whose drinking exceeded recommended drinking criteria had smaller total temporal volumes than a no/low drinking comparison group but that a greater lifetime number of drinks was associated with larger volume.

^e^ Phillips et al.^30^ reported greater drinking was associated with smaller total hippocampal volume and volume of one hippocampal subfield but with larger volume of two other hippocampal subfields.

^f^ Rossetti et al.^48^ reported that participants with AUD had smaller cerebellar volume than controls but among those with AUD, a greater number of monthly standard drinks was associated with larger volume.

### 3.1 Quality assessment

Twenty six of the studies were scored as ‘good’ quality, and one was scored as ‘fair’ with a score of 7/12 (8/12 was required for a ‘good’ rating). Thus, these studies can generally be considered high quality (see Table 3).

### 3.2 Prenatal alcohol exposure

One study examined prenatal exposure using data from the ABCD sample acquired when the participants were 9–10 years old. Children who were exposed in utero to varying amounts of alcohol (1–90 total drinks over the course of pregnancy), relative to those not exposed, had greater total cerebral volume and greater surface area in the occipital, parietal and temporal lobes.^27^ In this study, greater total number of maternal drinks during pregnancy was associated with greater total cerebral volume​​.^27^

### 3.3 Early adolescent alcohol consumption

The studies examining adolescent alcohol exposure and brain structure primarily used the NCANDA sample,^28^, ^29^, ^30^, ^31^, ^32^, ^33^, ^34^ but two studies used the IMAGEN^25^, ^35^ dataset. These studies generally compared adolescents with low or no alcohol use to those with moderate or heavy use. Some studies reported on global effects of alcohol consumption on brain structure and found that adolescents who reported greater levels of alcohol use showed smaller total grey matter volume and thickness than those with no/low use.^25^, ^32^, ^33^

Adolescents who reported greater levels of alcohol use also showed smaller volume in the prefrontal cortex,^35^ frontal lobe,^32^, ^33^ temporal cortex^35^ and parietal and temporal lobes, but not in the occipital lobe.^32^, ^33^ Several studies also reported on effects of alcohol in smaller parcels of the brain. Three studies found smaller volume and thickness in parcellations across the frontal lobe, including the middle frontal, superior frontal and inferior frontal gyri.^28^, ^29^, ^34^ They also reported smaller volume in the orbitofrontal cortex and precentral gyrus. In the parietal lobe, adolescents who had initiated heavier alcohol use showed smaller volume in the inferior parietal lobe, the precuneus and the postcentral gyrus.^29^ In the temporal lobe, one study reported similar thickness in adolescents who did or did not drink more alcohol in the inferior, middle and superior temporal gyri,^34^ but another paper showed smaller volume in these regions among heavier drinkers.^29^ Similarly, evidence suggested similar thickness in parcels of the occipital lobe^34^ but smaller volume in these parcels in adolescents with greater levels of alcohol use.^29^ Generally, alcohol use seemed to be associated with smaller volumes across the cortex in adolescents who report heavier alcohol use.

The effects of alcohol were mixed for the cingulate and insula, with some studies reporting greater volume or thickness in participants with heavier alcohol use in the insula,^33^ some reporting no difference in the anterior cingulate^34^ and insula^32^ and some reporting smaller volume in adolescents with heavier patterns of alcohol use in the insula^29^ and cingulate.^29^, ^33^ Greater alcohol exposure was associated with smaller hippocampal and amygdala volume.^30^ Few papers reported on the striatum. Greater alcohol exposure was associated with smaller total cerebellum volume.^31^ Among adolescents, subcortical volumes often demonstrated inconsistent effects (e.g., insula and hippocampus)^30^, ^32^ or were not reported (e.g., striatum). We suspect the inconsistencies may be due to adolescent alcohol exposure being highly variable and/or due to brain changes across development.

### 3.4 Low‐to‐moderate alcohol consumption in adulthood

The studies examining low‐to‐moderate exposure in adulthood came from three samples: the Framingham Offspring Study,^36^ HCP^37^, ^38^ and UK Biobank.^39^, ^40^, ^41^, ^42^, ^43^, ^44^, ^45^, ^46^ Unlike adolescent or heavy alcohol use studies that had an exposure and a comparison group, these studies generally had more than two groups, reflecting consumption patterns such as abstinence, low use (i.e., 0–7 standard drinks per week), moderate use, and heavier use. They often reported effects on total brain volume, and almost all indicated that, controlling for covariates such as sex, age, ethnicity, socioeconomic status and other substance use, greater alcohol consumption was associated with smaller brain volume.^36^, ^38^, ^39^, ^43^, ^45^, ^46^ Studies examining smaller parcellations of the brain reported that greater levels of alcohol use were associated with smaller volume and thickness in areas of the frontal, parietal, temporal and occipital lobes.^37^, ^45^, ^46^ Greater alcohol use was associated with smaller volume of the cingulate, insula, hippocampus, amygdala, putamen, nucleus accumbens, thalamus, cerebellum and brain stem.^39^, ^41^, ^42^, ^44^, ^45^, ^46^ These studies nearly unanimously reported that greater alcohol consumption was associated with smaller volume across nearly all brain regions.

### 3.5 Heavy alcohol consumption in adulthood

The studies examining heavy alcohol consumption (i.e., meeting criteria for alcohol use disorder or for binge drinking) in adulthood came from the ENIGMA consortium^25^, ^47^, ^48^, ^49^, ^50^, ^51^ and the HCP dataset.^52^ Participants included in the ENIGMA studies were mostly middle‐aged adults with a diagnosis of AUD and matched control adults, but the HCP sample represented young adults with frequent binge drinking, and some of the ENIGMA samples included younger adults. Some studies reported on global metrics of brain structure and found smaller grey matter volume^48^, ^49^, ^50^ and thickness^25^ across the cortex in the AUD group relative to the comparison sample. Individuals with AUD, relative to controls, showed less thickness in the middle and superior frontal gyrus, the orbitofrontal cortex and the precentral gyrus.^49^ In the parietal lobe, individuals with AUD, relative to controls, showed less thickness in the superior parietal lobule, the precuneus and the supramarginal gyrus.^49^ In the temporal lobe, individuals with AUD showed smaller volume and thickness in the superior temporal gyrus and less thickness in the inferior temporal gyrus, temporal pole and parahippocampal gyrus.^47^, ^49^, ^52^ In the occipital lobe, individuals with AUD showed less thickness in the fusiform gyrus and lateral occipital gyrus but no difference in the cuneus.^47^, ^49^

Heavy alcohol consumption also affected the limbic and subcortical structures. Individuals with AUD showed smaller volume and thickness of the insula and the anterior and posterior cingulate cortex than controls.^47^, ^49^, ^52^ Individuals with AUD had smaller volume in the amygdala, hippocampus, putamen, nucleus accumbens, pallidum and thalamus.^47^, ^48^, ^49^, ^50^, ^51^, ^52^ Counterintuitively, greater alcohol consumption in individuals with AUD was associated with larger cerebellar volume.^48^, ^52^

### 3.6 Sex as a biological variable

Half of the studies included in this review reported on the role of sex as a biological variable in the relationship between alcohol exposure and brain structure (see Table 3). Of the seven studies that included adolescents, the majority reported no moderating effect of sex on structural changes related to alcohol.^25^, ^29^, ^31^, ^34^, ^53^ One reported a small effect of sex, where females showed a greater decline in volume with increasing age in the supramarginal gyrus.^28^ Another, from the IMAGEN sample, found that, among adolescents who initiated heavier drinking, females showed greater atrophy than males.^35^ For studies of adults with low‐to‐moderate consumption, most papers found no moderation by sex.^37^, ^39^, ^41^, ^45^ One study, from the Framingham Offspring Study, showed that alcohol exposure was associated with greater volume loss in females than in males.^36^ One study was specifically designed to examine sex effects but only found moderation by sex in the cerebellum and amygdala; females showed a greater effect of alcohol towards volume loss in the cerebellum, and males showed a greater effect of alcohol towards volume loss in the amygdala.^48^ The study found that the effects of alcohol were larger, accounting for 3–9% reductions in volume, but the effects of sex were small and isolated.^48^ Another study showed that males with AUD, relative to healthy males, showed smaller volumes in the amygdala, but females with AUD did not differ from healthy females.^50^ Overall, the moderating effect of sex as a biological variable seems limited, but there were some indications that the deleterious effects of alcohol might be greater for female brains, except for the amygdala, which might be more susceptible in males.

## 4 DISCUSSION

This systematic review examined whether alcohol has different effects on brain structure depending on when during the lifespan the exposure or consumption occurs. The studies included in the present review were rated for quality (i.e., statistical rigour and study design), and all but one were determined to be in the highest category. All but one study reported correcting for multiple comparisons (see Table 3). Evidence from these generally high‐quality studies suggests that alcohol consumption leads to similar effects on regional patterns of brain volume in adolescence or adulthood. Further, low and moderate alcohol use showed effects on brain volume in the same direction and same areas as heavy use. Both adolescents and adults who reported heavier alcohol consumption, relative to those who did not, showed reduced frontal and parietal volumes. Alcohol consumption was associated with smaller volumes of the occipital and temporal cortices across developmental stages, but these effects were slightly less consistent. While studies of adults compared volume cross‐sectionally, the studies of adolescents often examined both cross‐sectional brain structure differences and differences in neurodevelopmental trajectories, such as the slope of change in volume over time. Given that 13 of the 27 studies included in the review statistically controlled for the use of other substances (such as tobacco, which may impact brain structure), we presume that the consistent effects on brain structure that emerged across these studies are attributable to the deleterious effects of alcohol itself, not merely to the concurrent use of other potentially harmful substances.

Some brain areas displayed different patterns by age, including the striatum, insula and cingulate. Specifically, adults with AUD or chronic, low‐level alcohol exposure had smaller volumes of these areas, but the effects were inconsistent in adolescents. It is possible that these areas may be sensitive to the chronic effects of alcohol but may be resilient in earlier developmental stages (see Table 4). Relatedly, it should be noted that there are little data on the effect of alcohol on the striatum in early adolescents; thus, suggestions about potential resilience of the striatum in this developmental stage are speculative.

Unlike adolescent and adult exposure, prenatal exposure was associated with larger brain volume and thickness in a sample of 9‐ to 10‐year‐olds. However, it is unclear if these effects will persist through later neural development. Adults with foetal alcohol spectrum disorders (FASD) have shown smaller volume in the striatum and other brain regions.^54^, ^55^ There is also evidence of regionally larger volume and thickness in adults with FASD. However, evidence was limited to a single study drawn from the ABCD sample, and alcohol exposure in the study was relatively low (i.e., participants reported an estimated range of total drinks consumed during pregnancy from 0–90, which is more in line with drinking patterns reported within the general population of pregnant individuals compared to prior studies that have specifically focused on FASD^56^). Alcohol exposure in the prenatal study was also confounded with sociodemographic variables, such as parental income and education, which also affect brain volume.^27^ Therefore, more evidence is needed to determine how the effects of prenatal alcohol exposure compare to adolescent or adult consumption.

While past neuroimaging studies often used voxel‐wise analytic approaches, recent evidence suggests that examining the brain in anatomically and functionally defined parcels is a more meaningful approach to neuroscientific questions.^57^ Neighbouring regions can differ in function, cell types, connectivity and topography.^58^ Parcellation study in humans long relied on post‐mortem analysis of architectonics (cell types, densities and distributions), but recent work with resting state functional connectivity has led to parcel boundary maps that align with existing architectonic maps and account for nearly 90% of the homogeneity in brain activation patterns during rest.^59^ These maps are highly reliable for individuals and generalize to new individuals not used for map development at the group level.^59^ Although none of the studies in this review applied these boundary maps for analysis of the effects of alcohol, they did use parcels that overlap with the boundary maps and offer some clues to which neural networks may be affected by alcohol. For example, evidence in adolescents and adults for lower volume in the superior and middle frontal gyri suggests impacts on three networks that span that cortical space: the fronto‐parietal, dorsal attention and default mode networks. Additionally, across adolescents and adults, the precuneus showed lower volume following alcohol consumption, implying change to the default mode network. This implication is corroborated by evidence that adults with AUD show lower efficiency in default mode network processing relative to controls.^60^ The patterns of smaller volume and thickness in individuals with heavier, relative to lighter, patterns of drinking suggest disruption to network efficiency that underlies healthy brain function.

These findings should be considered from a lifespan perspective. Typical brain development is characterized by a complex and time‐sensitive pattern of neurulation, neuronal proliferation and neural migration in utero,^61^ followed by rapid increases in grey and white matter volume and density during early childhood,^62^ and a period of cortical thinning during late childhood and adolescence, likely due to synaptic pruning.^5^ Brain volumes then slowly decline over the course of adulthood, with acceleration after roughly age 70^63^ with atrophy particularly pronounced in the frontal and temporal lobes.^64^ The effect of alcohol exposure or consumption during any of these stages must thus be considered relative to these typical trajectories. Taken together, the studies reviewed here suggest that alcohol exposure may accelerate or potentiate normative developmental trajectories, by increasing growth *in utero*, but accelerating volume loss during adolescence and adulthood. Although none of the studies included here focused specifically on older adults (i.e., over 65 years), a UK Biobank study of adults aged 45–80 that examined relative brain age to compare brain structure relative to peers of the same chronological age found that daily or near daily drinkers had a relative brain age almost half a year greater than peers of the same age who drank less frequently, suggesting that alcohol's effects are additive with those of ageing.^43^ Another study of adults aged 25 to 75 showed that AUD was associated with volume loss regardless of age but that adults with AUD did not show additional loss associated with earlier onset AUD or more lifetime drinks consumed.^65^ Thus, alcohol exposure at any stage of the lifespan may negatively impact brain structure, but the relationship may be non‐linear.

Our review suggests that pathologic alcohol use (i.e., AUD) is not necessary for volume loss to occur among people who drink alcohol. Specifically, Table 4 demonstrates that the pattern of effects (e.g., direction of effects and specific brain regions that show significant alcohol‐related volume or thickness loss) observed among mid‐to‐older adults who engaged in light‐moderate alcohol use and chronic alcohol use are similar. It is unknown whether there is a threshold of alcohol use that induces significant structural brain changes, but evidence from the UK Biobank^38^, ^39^, ^43^, ^45^, ^46^ and Framingham Offspring studies^36^ suggests that any increase in alcohol exposure (quantity or frequency) is associated with brain volume loss, with greater levels of alcohol use being associated with smaller brain volumes. Thus, even alcohol consumption consistent with current guidance in the United States, the United Kingdom and other nations (e.g., no more than 8–14 drinks per week for a male or 7–8 drinks per week for a female) may be sufficient to cause brain atrophy. Notably, reducing alcohol consumption could reverse some tissue loss. For example, among middle‐aged adults in AUD recovery (ranging in age from approximately 36–67 years), brain volume recovery has been shown to occur most quickly in the first month of abstinence and to be fastest among those who show the most alcohol‐related volume loss.^66^, ^67^

Overall, results from this systematic review indicate that alcohol affects brain structure similarly across developmental stages. Such structural changes could reflect neuronal loss caused by cellular effects of alcohol exposure, such as oxidative stress that can result from proinflammatory cytokines released in response to alcohol.^8^ The tissue loss may depend on the amount of duration of alcohol exposure, but studies have estimated that the structural changes have effect sizes in the small to medium range, accounting for 3–9% reductions in volume.^48^ In fact, the magnitude of the differences in brain volume between individuals with AUD relative to controls is large enough that a trained radiologist can visually observe brain scans and classify whether a person has AUD with 66% accuracy, and machine learning approaches are even more accurate than humans, achieving up to 73% accuracy.^68^ The loss of brain tissue seems to relate to poorer neurocognitive performance related to heavy alcohol use.^69^ These negative effects of alcohol on brain structure and function underscore the need for early, targeted clinical interventions, as well as for treatment approaches tailored to developmental stage.

### 4.1 Limitations of the literature

One challenge within this literature is the inconsistency in reporting and analysis of brain changes. Some studies used fine‐grained parcellations of the brain and did not report on whole brain metrics or larger patterns, while other studies primarily reported on whole brain effects or large parcellations and may have missed smaller effects. The discrepancies between these two approaches present a challenge for drawing inference across studies since it is unclear if the approach affects the results. It would be helpful for studies that use fine grained parcellations to also report whole brain metrics, and vice versa. Further, the variable methods used to quantitate brain morphometrics across studies may also contribute to the variability of findings across studies. The literature also has inconsistencies in how it deals with potential factors that could influence the effects of alcohol, such as biological sex, use of other substances, sociodemographic variables such as education and health status (e.g., diabetes). For example, 13 of the 27 studies included in this review covaried for the use of other substances (such as cigarette smoking, which is known to impact brain structure), but the lack of consideration of other substance use in the other papers is a notable limitation. In addition, health, socioeconomic status, race and ethnicity are all potentially linked and may all moderate the effects of alcohol, so careful consideration will be required to disentangle the effects of each. We acknowledge that it is possible that changes in brain structure among adults with AUD may be related to the potential confounding factors listed above, not simply to greater consumption of alcohol or duration of drinking. Relatedly, when relating volume effects to age, most studies do not correct for duration of drinking. Notably, the UK Biobank included only people with British ancestry, so addressing some of these questions may require new data collection.

An additional limitation is that we confined this review to seven neuroimaging consortium studies (with an alcohol‐exposed sample size of at least 100 individuals), and our focus on only these seven consortia may mean that other potentially informative studies were excluded. Also, in this review, only studies that tested the effects of alcohol use on brain volume, cortical thickness or surface area were included, but it is also likely that the reverse is true: that is, that pre‐existing brain volume (or thickness or surface area) differences may be associated with alcohol use.^70^, ^71^ While the evidence largely supports that alcohol exposure has a causal role in changing brain volume, this does not negate the possibility that brain volume could influence future alcohol use.

A final important limitation is that papers based on data from a given consortium may have had some overlap in the samples that were included, but the extent of overlap is not possible to determine from the papers. This may be considered a weakness in the methodology, given that the consistency of these findings may have been overestimated as a function of this overlap, as the same subjects may have been included in multiple papers and produced the same outcomes. However, a strength of this method comes from the fact that different analyses conducted on these potentially overlapping samples across papers produced consistent results, thereby increasing our confidence in the overall patterns that emerged from these analyses. Specifically, although there was some overlap in samples, research questions and analytic techniques differed meaningfully between papers reporting on the same consortium. For example, of the papers that analysed the NCANDA sample, one focused specifically on the amygdala and hippocampus,^30^ one focused on the cerebellum,^31^ one used machine learning to explore cortical thickness,^34^ several specifically explored longitudinal changes^28^, ^29^ and developmental trajectories,^31^, ^32^ while another was cross‐sectional.^33^ Of the papers that analysed the ENIGMA sample, one paper focused on gender differences across many ROIs,^48^ one focused specifically on sex differences in the amygdala and hippocampus,^50^ one used structural covariance networks^25^ and one used a support vector machine classification approach.^49^

Finally, it should be noted that this review only included one study on prenatal alcohol exposure (as only one study met our inclusion criteria), which limits the conclusions that can be drawn from this review about the role of prenatal alcohol exposure on brain structure.

### 4.2 Summary and future directions

Data from high quality, large‐scale, multi‐site neuroimaging studies indicate that alcohol exposure and consumption deleteriously affect brain volume throughout development. The reliability of structural MRI measurement of brain volume is excellent, with intraclass correlation greater than 0.9,^72^ so these effects should be considered strong evidence. However, despite these robust data, many critical questions remain unanswered. High‐quality longitudinal samples followed through adolescence, early adulthood and beyond are needed. The strongest data to date on the effects of adolescent alcohol consumption come from the NCANDA study, which enrolled more participants than any previous adolescent neuroimaging study but remained limited by the number of youths who initiated heavy consumption within the follow‐up period. The ongoing ABCD Study, with a larger initial enrolment (*N* = 11 880) and a longer follow‐up period, will further refine our understanding of the neural effects of adolescent and young adult alcohol consumption. Evidence to date is compelling that, relative to lower levels of alcohol use, higher levels of alcohol exposure or consumption across the lifespan negatively affect the brain, with the caveats that older age (convolved with longer drinking duration) and more severe AUD (accompanied by potentially more numerous or severe comorbidities) may be associated with greater brain volume deficits.

## AUTHOR CONTRIBUTIONS

Hollis C. Karoly, Katelyn T. Kirk‐Provencher, Joseph P. Schacht and Joshua L. Gowin were responsible for the conceptualization of the review and writing different sections of the manuscript. All authors critically reviewed the content and approved the final version for publication.

## CONFLICT OF INTEREST STATEMENT

The authors have no conflicts of interest.

## Supporting information


**Table S1.** Description of Large‐Scale Studies and Consortiums Included in this Review.
**Table S2.** Group Comparisons of Brain Regions Affected Across the Lifespan Due to Alcohol Exposure

## Data Availability

Data sharing is not applicable to this article, as no new data were created or analysed in this study.
