# Supplementary material for: Alcohol and brain structure across the lifespan: A systematic review of large‐scale neuroimaging studies
Source: Addict Biol. 2024 Sep 24;29(9):e13439. doi: 10.1111/adb.13439 (PMC11421948; doi:10.1111/adb.13439)
Supplement: Supplementary file 1 — Table S1. Description of Large‐Scale Studies and Consortiums Included in this Review. Table S2. Group Comparisons of Brain Regions Affected Across the Lifespan Due to Alcohol Exposure [file ADB-29-e13439-s001.docx]

**Supplemental Table 1**. Description of Large-Scale Studies and Consortiums Included in this Review.

| **Study Abbreviation** | **Description** | **Examples of Structural Brain Imaging & Variable Measures** | **Drinking Definition** | **Corresponding Alcohol**  **Exposure Timeframe** |
| --- | --- | --- | --- | --- |
| ABCD | The Adolescent Brain Cognitive Development Study, established in 2015, led by the ABCD Research Consortium has enrolled children (ages 9-10) across 21 research sites throughout the United States of America. Participants are followed through adolescence and young adulthood to examine how various childhood experiences, including substance use, impact brain development. Neuroimaging conducted at baseline and two- and four-year follow-ups. Website: <https://abcdstudy.org/> | - Structural MRI (3D T1 – Weighted; 3D T2 – Weighted) - Prenatal exposure by trimester to medications, alcohol, tobacco, and drugs (parent report) - Medications and substances used during breastfeeding (parent report) - Household Substance Use, Density, Storage & Second-Hand Exposure (parent report) - Sibling substance use - Diagnostic Interview for DSM-5 Alcohol Use Disorder/Drug Use Disorder modules - Alcohol breathalyzer screen - Substance and environmental toxin exposure (baby teeth) - Substance use metabolites (hair strands) - Heard of alcohol (Peer Group Deviance, Perceived Harm of Substance Use, Peer Tolerance of Use) - Having tried alcohol – even a sip (iSay II Q2 Sipping Items) - Having ever used alcohol – a full drink (Timeline Follow-Back Survey, Acute Subjective Response to Alcohol) - Having two or more occasions of alcohol use (Hangover Symptom Scale, Rutgers Alcohol Problem Index, Drinking Motives Questionnaire) | - Abstinent = < 1 standard drink/occasion throughout pregnancy - Light = 1-2 drinks/occasion, <7   drinks/week   - Moderate = 3-4 drinks /occasion, <7 drinks /week) - Heavy = <5 drinks /occasion, 7+ drinks/week) - Binge = 5+ drinks per occasion - Light reducers = light before knowing of pregnancy, abstinent after knowing of pregnancy - Light, stable users = Light before and after knowing of pregnancy - Heavier reducers = Moderate, heavy, and binge drinkers before knowing of pregnancy, abstinent or light drinking after knowing of pregnancy - Heavy stable users = Heavy before and after knowing of pregnancy   Note. The only ABCD study included in this review analyzed prenatal exposure. The definitions above are from that study. | Prenatal |
| ENIGMA | The Enhancing Neuro Imaging Genetics through Meta Analysis Consortium, established in 2009, is an international consortium of 50 working groups comprised of imaging genomic, neurology, and psychiatry researchers, with the goal of better understanding brain structure and function in various patient populations, including those with substance use disorders. Researchers share neuroimaging data and methods to create large-sample databases. The ENIGMA-Addiction working group has compiled imaging data representing more than 14,000 participants. Website: <https://enigma.ini.usc.edu/> | - Structural MRI - Alcohol use (ever used, age of first use, heavy drinking days, and daily, weekly, and past year use) - Alcohol Use Disorder (lifetime diagnosis, remission status, duration of abstinence) - Alcohol Use Disorder Identification Test, - Substance use Risk Profile Scale - Structured Clinical Interview for DSM | - Alcohol dependent (DSM-IV criteria) - Not alcohol dependent (DSM-IV criteria) | Heavy Adult Exposure |
| FOS | The Framingham Offspring Study, established in 1971, consists of the offspring (ages 5-70) of the original Framingham Heart Study cohort from Framingham, Massachusetts, United States of America. The study examines risk factors of cardiovascular disease through physical exams, blood samples, and self-report lifestyle data, and began including brain MRI scans in 1999. Website: <https://www.framinghamheartstudy.org/> | - Brain MRI (1.0T and 1.5T T2 – Weighted; 3D T1 – Weighted) - Alcohol use (quantity and frequency of different types of alcohol, e.g., beer, wine, liquor used per week and month, age stopped drinking, days drank per year, maximum number of drinks in 24 hours, binge drinking) | - Abstainers = No current drinking - Former drinkers = based on drinking status at earlier exams - Low = 1-7 drinks week - Moderate = 8-14 drinks per week - High = 15 or more drinks per week | Low to Moderate Adult Exposure |
| HCP | The Human Connectome Project, established in 2010, maps the brain to connect human behavior to neural structures and functions the project has collected data from healthy young adults (ages 21-35), and across the lifespan (prenatal, ages 0-5, 6-21, and 36-100+). Website: <https://www.humanconnectome.org/> | - Brain MRI (3T MR, 7T MR, magnetoencephalography) - Alcohol breathalyzer - Alcohol use (NIDA Substance Abuse and Alcohol Core [ages 12-21 and parents], Semi-Structed Assessment for the Genetics of Alcoholism, age at first use, binge drinking, past 12-month frequency and quantity) - DSM-IV alcohol abuse and alcohol dependence criteria - Parent drug and alcohol problems - 7-day retrospective alcohol use (total drinks, days drank, weekday and weekend drinks for beer, wine, hard liquor, malt liquor, and other alcohol | Heavy drinking categories:   - Binge drinkers = 4 or more drinks for women and 5 or more drinks for men in single day - Non-binge drinkers   Low-moderate drinking categories:   - 1 = never - 2 = 1–11 days/year - 3 = 13 days/month - 4= weekly or greater | Heavy Adult Exposure  Low to Moderate Adult Exposure |
| IMAGEN | The IMAGEN Study, established in 2010, is a European study investigating biological, psychological, and environmental factors associated with adolescent brain development. The study enrolled adolescents (at age 14) and their parents from eight sites across four European Union countries., Adolescents were followed through age 22. Data collected included brain imaging, psychiatric, and genetic variables. Website: <https://imagen-project.org/> | - MRI - Substance Use Risk Profile Scale (child and parent report) - Alcohol Use Disorder Identification Test (child and parent report) - European School Survey Project on Alcohol and Drugs (child and parent report) - Michigan Alcoholism Screening Test (child and parent report) - Rutgers Alcohol Problem Index - Drinking Motives Questionnaire - Structured Clinical Interview for DSM (Substance Use Disorder modules) - Development and Well-Being Assessment Interview - Timeline Follow-Back Interview - Genetic Screening and Family History of Psychiatric Disorders Interview (parent report) - Pregnancy and Birth Questionnaire (parent report) | - Hazardous drinkers (AUDIT   score >= 8)   - Non-hazardous drinkers (AUDIT score <= 7) | Adolescent Exposure |
| NCANDA | The National Consortium on Alcohol and Neurodevelopment in Adolescence consortium, established in 2012, consists of five sites across the United States of America. The study enrolled high-risk participants (ages 12-21; 692 “no/low drinkers, 139 “drinkers”) and followed them for three years. The study investigates the effects of alcohol use, related consequences, and mental health symptoms on adolescent brain development and risk for developing an alcohol use disorder. Website: <http://www.ncanda.org/> | - MRI (3T T1-Weighted) - Alcohol use (lifetime drinking days, maximum drinks on one occasion, Hangover Symptoms Survey, Alcohol Expectancy Questionnaire, Customary Drinking and Drug Use Record, Peer Group Deviance) - Family history (child and parent report of Family History Assessment Module, Computerized Semi-Structured Assessment for the Genetics of Alcoholism) - Achenbach System of Empirically Based Assessments (child and parent report) - Access to Substances & Neighborhood Strength (child and parent report) | - No/Low Drinking = reported no or low quantity and frequency consumption (e.g., <1x/month, <2 drinks on average, and <4 drinks maximum). - Moderate Drinking = ranged from low drinking frequency (e.g., <1x/month) with moderate quantity consumption (e.g., with 2-3 drinks on average and 4-5 drinks maximum) to moderate frequency (e.g., 1x/week) and low quantity consumption (e.g., with 2 drinks on average and <4 drinks maximum). - Heavy Drinking = Ranged from moderate frequency (e.g., 2x/month) with high quantity consumption (e.g., with 3-4 drinks on average and > 4 drinks maximum) to higher frequency (e.g., 1x/week or more) with moderate quantity consumption (e.g., with 2-3 drinks on average and >4 drinks maximum). | Adolescent Exposure |
| UK Biobank | The UK Biobank established in 2006, is a large-scale prospective study biomedical database of half a million individuals (ages 40-69) in the United Kingdom. The study aims to better understand the development of common and life-threatening conditions through the ongoing collection of biomedical samples, health records, and lifestyle information, including alcohol use. Beginning in 2014, the study aimed to conduct whole-body MRI scans, including to assess brain morphology, on 100,000 participants and 60,000 were scanned by 2022. Website: <https://www.ukbiobank.ac.uk/> | - MRI - Alcohol use (quantity and frequency, specific beverages, reasons for recent stopping) - Genetic and biological data (blood, saliva, urine) | - Abstainers = 0 drinks per week - Light drinkers = Up to 8.4 drinks per week - Moderate drinkers = > 8.4 – 15.4 drinks per week - Heavy drinkers > 15.4 drinks per week | Low to Moderate Adult Exposure |

**Supplemental Table 2.** Group Comparisons of Brain Regions Affected Across the Lifespan Due to Alcohol Exposure

| **Brain Structure** | **Alcohol Exposure Timeframe** | | | |
| --- | --- | --- | --- | --- |
|  | Prenatal exposure: Children & Adolescents^a^ | Adolescence & early adulthood: Initiation and use^b^ | Mid- to older- adulthood: Light to moderate use^b^ | Mid- to older- adulthood: Chronic use^b^ |
| **Global Measurements** | | | | |
| Whole Brain | | | | |
| Total cerebral volume | ↑ (Lees et al., 2020) |  | ↓ (Evangelou 2021)  ↓ (Daviet 2022; Paul 2008)  ↓ (Ning 2020) | ↓ (Rossetti 2021) |
| Gray Matter | | | | |
| Total volume/thickness | ↑ (Lees et al., 2020) volume,  ≈ (Lees et al., 2020) thickness | ↓ (Pfefferbaum 2016) volume  ↓ (Pfefferbaum et al., 2018) volume  ↓ thickness (Ottino Gonzalez 2022) | ↓ volume (Topiwala 2022)  ↓volume (Daviet 2022)  ↓ volume (Evangelou 2021)  ≈ volume (Zhao 2021)  ↓ thickness (Zhao 2021) | ↓ volume (Grace 2021, Rossetti 2021)  ↓ thickness (Ottino Gonzalez 2022) |
| **Frontal Lobe** | | | | |
| Total Frontal |  | ↓ volume (pfefferbaum 2016)  ↓ volume (Pfefferbaum et al., 2018) | ↓ volume (Topiwala 2022)  ↓ volume (Daviet 2022) |  |
| Prefrontal Cortex (PFC) | | | | |
| Total PFC |  | ↓ volume (Robert 2020) | ↓ thickness (Morris 2019) |  |
| Frontal Gyri | | | | |
| medial frontal gyrus |  |  |  |  |
| middle frontal gyrus | ↑ area (Lees et al., 2020)  ↑ volume (Lees et al., 2020) | ↓ thickness (Sun 2023)  ↓ volume (Luo 2022, Infante 2022)  ↓ volume (Pfefferbaum 2018) |  | ↓ thickness (Mackey 2019)  ≈ thickness (Navarrri 2022) |
| superior frontal gyrus | ↑ area (Lees et al., 2020)  ↑ volume (Lees et al., 2020) | ↓ thickness (Sun 2023)  ↓ volume (Infante 2022)  ↓ volume (Pfefferbaum et al., 2018) |  | ↓ thickness (Mackey 2019; Navarri 2022) |
| inferior frontal gyrus | ↑ area (Lees et al., 2020)  ↑ volume (Lees et al., 2020) | ≈ thickness (Sun 2023)  ↓ volume (Luo 2022, Infante 2022) | ↓ thickness (Morris 2019) | ≈ thickness (Navarrri 2022) |
| Orbitofrontal Cortex | ↑ area (Lees et al., 2020)  ↑ volume (Lees et al., 2020) | ↓ volume (Infante 2022) | ↓ thickness (Morris 2019)  ↓ volume (Evangelou 2021)  ↓ volume (Zhao 2019)^****^ | ≈ volume (Rossetti 2021)  ↓ thickness (Mackey 2019)  ≈ thickness (Navarrri 2022) |
| Precentral Lobule/Gyrus | ↑ area (Lees et al., 2020)  ↑ volume (Lees et al., 2020) | ↓ volume (Infante 2022) | ↓ thickness (Morris 2019)  ↓ volume (Topiwala 2022)  ↓ volume (Zhao 2019)^****^ | ↓ thickness (Mackey 2019)  ≈ thickness (Navarrri 2022) |
| Paracentral Lobule/Gyrus |  | ↓ volume (Infante 2022) | ↓ volume (Topiwala 2022)  ↓ volume (Zhao 2019)^****^ | ↓ thickness (Mackey 2019)  ≈ thickness (Navarrri 2022) |
| Frontal Pole | ↑ area (Lees et al., 2020)  ↑ volume (Lees et al., 2020) | ↓ volume (Infante 2022) |  | ≈ thickness (Navarrri 2022) |
| **Parietal Lobe** | | | | |
| Total Parietal |  | ↓ thickness (pfefferbaum 2016)  ≈ volume (Pfefferbaum et al., 2018) | ↓ volume (Topiwala 2022)  ↓ volume (Daviet 2022) |  |
| Superior Parietal Lobe | ↑ area (Lees et al., 2020)  ↑ volume (Lees et al., 2020) | ≈ thickness (Sun 2023)  ↓ volume (Infante 2022) |  | ↓ thickness (Mackey 2019)  ≈ thickness (Navarrri 2022) |
| Inferior Parietal Lobe | ↑ area (Lees et al., 2020)  ↑ volume (Lees et al., 2020) | ↓ volume (Infante 2022) | ↓ thickness (Matloff 2020)  ↓ volume (Zhao 2019)^****^ | ≈ thickness (Navarrri 2022) |
| Precuneus | ↑ area (Lees et al., 2020)  ↑ volume (Lees et al., 2020) | ↓ volume (Infante 2022) | ↓ thickness (Morris 2019) | ↓ thickness (Mackey 2019)  ≈ thickness (Navarrri 2022) |
| Postcentral Gyrus | ↑ area (Lees et al., 2020)  ↑ volume (Lees et al., 2020)  ↑ thickness ^15^ | ≈ thickness (Sun 2023)  ↓ volume (Infante 2022) | ↓ thickness (Morris 2019)  ↓ volume (Topiwala 2022) | ≈ thickness (Navarrri 2022) |
| Supramarginal | ↑ area (Lees et al., 2020)  ↑ volume (Lees et al., 2020) | ≈ thickness (Sun 2023)  ↓ volume (Luo 2022, Infante 2022) |  | ↓ thickness (Mackey 2019)  ≈ thickness (Navarrri 2022) |
| **Temporal Lobe** | | | | |
| Total Temporal |  | ↑, ↓volume (Pfefferbaum 2016)^*^  ↓ volume (Robert 2020)  ≈ volume (Pfefferbaum et al., 2018) | ↓ volume (Topiwala 2022)  ↓ volume (Daviet 2022) |  |
| Entorhinal Cortex |  | ≈ volume (Infante 2022) |  | ≈ thickness (Navarrri 2022) |
| Middle Temporal Gyrus | ↑ area (Lees et al., 2020)  ↑ volume (Lees et al., 2020)  ↑ thickness (Lees et al., 2020) | ≈ thickness (Sun 2023)  ↓ volume (Infante 2022) | ↓ thickness (Matloff 2020)  ↓ thickness (Morris 2019)  ↓ volume (Zhao 2019)^****^ | ≈ thickness (Navarrri 2022) |
| Superior Temporal Gyrus | ↑ volume (Lees et al., 2020) | ≈ thickness (Sun 2023)  ↓ volume (Luo 2022, Infante 2022) | ↓ thickness (Matloff 2020)  ↓ thickness (Morris 2019)  ↓ volume (Zhao 2019)^****^ | ↓ volume (Li 2021)  ↓ thickness (Mackey 2019)  ≈ thickness (Navarrri 2022) |
| Inferior Temporal Gyrus | ↑ area (Lees et al., 2020)  ↑ volume (Lees et al., 2020) | ≈ thickness (Sun 2023)  ↓ volume (Infante 2022) | ↓ thickness (Matloff 2020) | ↓ thickness (Mackey 2019; Navarri 2022) |
| Superior Temporal Sulcus | ↑ area (Lees et al., 2020)  ↑ volume (Lees et al., 2020) | ↓ volume (Infante 2022) |  | ≈ thickness (Navarrri 2022) |
| Temporal Pole | ↑ volume (Lees et al., 2020) | ≈ volume (Infante 2022) |  | ↓ thickness (Mackey 2019; Navarri 2022) |
| Transverse Temporal Gyrus | ↑ area (Lees et al., 2020)  ↑ volume (Lees et al., 2020) | ≈ volume (Infante 2022) |  | ≈ thickness (Navarrri 2022) |
| Parahippocampal Gyrus | ↑ volume (Lees et al., 2020) | ≈ thickness (Sun 2023)  ≈ volume (Infante 2022) |  | ↓ thickness (Mackey 2019, Navarrri 2022) |
| **Occipital Lobe** | | | | |
| Total Occipital |  | ≈ volume (Pfefferbaum 2016)  ≈ volume (Pfefferbaum 2018) | ↓ volume (Topiwala 2022)  ↓ volume (Zhao 2019)^****^ |  |
| Lateral Occipital | ↑ area (Lees et al., 2020)  ↑ volume (Lees et al., 2020)  ↑ thickness (Lees et al., 2020) | ↓ volume (Infante 2022) | ↓ thickness (Morris 2019) | ↓ thickness (Mackey 2019)  ≈ thickness (Navarrri 2022) |
| Fusiform Gyrus | ↑ area (Lees et al., 2020)  ↑ volume (Lees et al., 2020) | ↓ volume (Luo 2022, Infante 2022) |  | ↓ thickness (Mackey 2019; Navarri 2022) |
| Occipital Sulcus |  |  |  |  |
| Cuneus | ↑ volume (Lees et al., 2020)  ↑ thickness (Lees et al., 2020) | ≈ thickness (Sun 2023)  ↓ volume (Infante 2022) |  | ≈ thickness (Navarrri 2022) |
| Lingual | ↑ area (Lees et al., 2020)  ↑ volume (Lees et al., 2020)  ↑ thickness (Lees et al., 2020) | ≈ thickness (Sun 2023)  ↓ volume (Infante 2022) | ↓ volume (Topiwala 2022) | ≈ thickness (Navarrri 2022) |
| Calcarine |  | ≈ thickness (Sun 2023)  ↓ volume (Infante 2022) |  | ≈ thickness (Navarrri 2022) |
| **Subcortical and Limbic Structures** | | | | |
| Cingulate | | | | |
| Total cingulate |  | ↓ thickness (Pfefferbaum 2016),  ↓ volume (Pfefferbaum, 2018) | ↓ volume (Evangelou 2021)  ↓ volume (Daviet 2022) |  |
| Anterior | ↑ area (Lees et al., 2020)  ↑ volume (Lees et al., 2020) | ≈ thickness (Sun 2023)  ↓ volume (Infante 2022) | ↓ thickness (Morris 2019)  ↓ volume (Zhao 2022)  ↓ volume (Zhao 2019)^****^ | ↓ volume (Li 2021)  ↓ thickness (Mackey 2019; Navarri 2022) |
| Middle |  | ≈ thickness (Sun 2023) | ↓ volume (Zhao 2019)^****^ |  |
| Posterior | ↑ area (Lees et al., 2020)  ↑ volume (Lees et al., 2020) | ≈ thickness (Sun 2023)  ↓ volume (Pfefferbaum 2018) | ↓ thickness (Matloff 2020)  ↓ thickness (Morris 2019) | ↓ thickness (Li 2021)  ↓ thickness (Mackey 2019, Navarrri 2022) |
| Isthmus | ↑ area (Lees et al., 2020)  ↑ volume (Lees et al., 2020) | ≈ volume (Infante 2022) |  | ≈ thickness (Navarrri 2022) |
| Median |  |  |  |  |
| Paracingulate gyrus |  |  |  | ↓ volume (Li 2021) |
| Hippocampus | ↑ volume (Lees et al., 2020) | ↑,↓ volume (Phillips 2021)^**^  ↓ volume (Phillips 2021) | ↓ volume (Matloff 2020)  ↓ volume (Hedges 2019) | ↓ volume (Chye 2020)  ↓ volume (Navarri 2022)  ↓ volume (Grace 2021; Mackey 2019; Navarri 2022; Rossetti 2021) |
| Insula |  | ≈ thickness (Sun 2023)  ↑ thickness (Pfefferbaum 2016),  ↓ volume (Infante 2022)  ≈ volume (Pfefferbaum 2018) | ↓ thickness (Morris 2019)  ↓ volume (Evangelou 2021)  ↓ volume (Daviet 2022) | ↓ volume (Li 2021)  ↓ thickness (Mackey 2019)  ≈ thickness (Navarrri 2022) |
| Claustrum |  |  |  |  |
| Amygdala | ↑ volume (Lees et al., 2020) | ↑ volume (Phillips 2021)  ↓ volume (Phillips et al., 2021) | ≈ volume (Topiwala 2022)  ↓ volume (Zhao 2022)  ↓ volume (Daviet 2022) | ↓ volume (li 2021)  ↓ volume (Chye 2020)  ↓ volume (Grace 2021; Mackey 2019; Navarri 2022; Rossetti 2021) |
| Striatum | | | | |
| Total striatum |  |  |  |  |
| Putamen |  |  | ↓ volume (Daviet 2022) | ↓ volume (Chye 2020)  ↓ volume (Navarri 2022)  ↓ volume (Mackey 2019; Rossetti 2021) |
| Nucleus accumbens |  |  | ↓ volume (Zhao 2022) | ≈ volume (Rossetti 2021)  ↓ volume (Mackey 2019; Navarri 2022)  ↓ volume (Chye 2020) |
| Caudate |  |  |  | ≈ volume (Chye 2020)  ≈ volume (Rossetti 2021, Navarrri 2022) |
| Pallidum |  |  | ↑ volume (Daviet 2022) | ≈ volume (Chye 2020)  ↓ volume (Rossetti 2021)  ≈ volume (Navarrri 2022) |
| Thalamus | ↑ volume (Lees et al., 2020) |  | ↓ volume (Evangelou 2021)  ↓ volume (Topiwala 2022) | ↓ volume (Chye 2020)  ↓ volume (Navarri 2022)  ↓ volume (Mackey 2019; Navarri 2022; Rossetti 2021) |
| Ventral Diencephalon | ↑ volume (Lees et al., 2020) |  |  |  |
| **Cerebellum** | | | | |
| Total Cerebellum | ↑ volume (Lees et al., 2020) | ↓ volume (Sullivan 2020) | ↓ volume (Topiwala 2022) | ↑ volume (Li 2021, Rossetti 2021)***  ↓ volume (Rossetti 2021)*** |
| **Brain Stem** | | | | |
| Pons |  |  | ↓ volume (Topiwala 2022)  ↓ volume (Daviet 2022) |  |

*Note.* References for each finding are indicated by superscript numerical citations. ↑ and ↓ indicate alcohol exposure was associated with greater or smaller volume/thickness/surface area, respectively, ≈ indicates the reported absence of a significant effect of alcohol exposure, and empty cells indicate that effects or lack of effects were not reported in the studies included in this review.

^a^ Reported results indicate comparisons between children or adolescents with prenatal alcohol exposure relative to those without prenatal exposure.

^b^ Reported results indicate comparisons between individuals (i.e., adolescents, young adults, or older adults) who drink alcohol (i.e., initiation of alcohol use, light, moderate, or chronic alcohol use) relative to individuals who do not drink alcohol/drink low amounts.

^*^ Pfefferbaum et al. reported that participants whose drinking exceeded recommended drinking criteria had smaller total temporal volumes than a no/low drinking comparison group, but that a greater lifetime number of drinks was associated with larger volume.

^**^ Phillips et al. reported greater drinking was associated with smaller total hippocampal volume and volume of one hippocampal subfield, but with larger volume of two other hippocampal subfields.

^***^ Rosetti et al reported that participants with AUD had smaller cerebellar volume than controls, but amongst those with AUD, a greater number of monthly standard drinks was associated with larger volume.

^****^ Zhao et al (2019) reported widespread decreases in volume associated with greater alcohol consumption, but they applied a stringent correction for multiple comparisons (q < 1.72E-4). For completeness, we report the regions they list as showing a relationship, given that their correction is more conservative than the typical paper in this literature.

**Complete Search Terms for All Databases**

**Web of science search**

TS=(Ethanol OR alcohol* OR AUD OR “heavy drinking” OR “binge drinking” OR "teen drinking" OR "teenage drinking" OR "adolescent drinking" OR "youth drinking" OR intoxicat* OR beer OR wine)

AND TS=(brain OR cereb* OR “gray matter” OR “surface area” OR cortic* OR cortex)

AND TS=(“magnetic resonance imaging” OR MRI OR neuroimag*) AND

TS=(“Adolescent Brain Cognitive Development Study” OR ABCD OR “Enhancing Neuro Imaging Genetics through Meta Analysis” OR ENIGMA OR FOS OR “Framingham Offspring Study” OR “Human Connectome Project” OR HCP OR IMAGEN OR “National Consortium on Alcohol and Neurodevelopment in Adolescence” OR NCANDA OR “UK Biobank”) OR FG=(“U24 DA041147” OR U24DA041147 OR “U54 EB020403” OR U54EB020403 OR N01-HC-25195 OR N01HC25195 OR U54MH091657 OR U01AG052564 OR “U54 MH091657” OR “U01 MH109589” OR “U01 AG052564” OR MRF_MRF-058-0009-RG-DESR-C0759 OR “U24 AA021695” OR MC_QA137853 OR MC_PC_17228)

**PUBMED SEARCH**

**Concept 1**

**"Alcoholic Intoxication"[Mesh] OR Alcoholism[Mesh] OR "Alcohol Drinking"[Mesh] OR Ethanol[Mesh] OR alcohol*[tiab] OR AUD[tiab] OR "heavy drinking"[tiab] OR "binge drinking"[tiab] OR "teen drinking"[tiab] OR "teenage drinking"[tiab] OR "adolescent drinking"[tiab] OR "youth drinking"[tiab] OR “juvenile drinking”[tiab] OR intoxicat*[tiab] OR ethanol[tiab] OR beer[tiab] OR wine[tiab]**

**Concept 2**

brain[Mesh] OR brain[tiab] OR cereb*[tiab] OR “gray matter”[tiab] OR “surface area”[tiab] OR cortic*[tiab] OR cortex[tiab]

**Concept 3**

"Magnetic Resonance Imaging"[Mesh] OR "Neuroimaging"[Mesh] OR “magnetic resonance imag*”[tiab] OR MRI[tiab] OR neuroimag*[tiab] OR brain[Mesh] OR brain[tiab] OR cereb*[tiab] OR “gray matter”[tiab] OR “surface area”[tiab] OR cortic*[tiab] OR cortex[tiab]

Potentially either remove Concept 2 or combine Concept 2 & 3 together with OR to make the search more sensitive.

**Consortia aka Concept #4**

“Adolescent Brain Cognitive Development Study” [tw] OR ABCD *[tw]*

OR U24 DA041147 *[gr*] OR U24DA041147 *[gr*] OR “Enhancing Neuro Imaging Genetics through Meta Analysis” *[tw]*  OR ENIGMA *[tw]*  OR **U54 EB020403** *[gr]* OR **U54EB020403** *[gr]* OR “Framingham Offspring Study” [*tw]*  OR FOS *[tw]*  OR N01-HC-25195 *[gr]* OR N01HC25195 *[gr]* OR “Human Connectome Project” *[tw]*  OR HCP *[tw]*  OR U54MH091657 *[gr]* OR U01AG052564 *[gr]* OR U54 MH091657 *[gr]* OR U01 MH109589 *[gr]* OR U01 AG052564 *[gr]* OR IMAGEN *[tw]*  OR [MRF_MRF-058-0009-RG-DESR-C0759](https://pubmed.ncbi.nlm.nih.gov/?sort=date&term=MRF_MRF-058-0009-RG-DESR-C0759%2FMRF%2FMRF%2FUnited+Kingdom%5BGrant+Number%5D&sort_order=desc) *[gr]* OR “National Consortium on Alcohol and Neurodevelopment in Adolescence” *[tw]*  OR NCANDA *[tw]*  OR U24 AA021695 *[gr]* OR “UK Biobank” *[tw]*  OR MC_QA137853 *[gr]* OR MC_PC_17228 *[gr]*

**Embase Search (which includes MEDLINE)**

('alcoholic intoxication'/exp OR 'alcoholism'/exp OR 'alcohol drinking'/exp OR 'ethanol'/exp OR (alcohol* OR aud OR 'heavy drinking' OR 'binge drinking' OR 'teen drinking' OR 'teenage drinking' OR 'adolescent drinking' OR 'youth drinking' OR intoxicat* OR ethanol OR beer OR wine):ti,ab,kw) AND ('brain'/exp OR (brain OR cereb* OR 'gray matter' OR 'surface area' OR cortic* OR cortex):ti,ab,kw) AND ('neuroimaging'/exp OR 'magnetic resonance imaging'/exp OR ('magnetic resonance imaging' OR mri OR neuroimag*):ti,ab,kw) AND 'adolescent brain cognitive development study' OR ABCD OR 'U24 DA041147' OR U24DA041147 OR 'enhancing neuro imaging genetics through meta-analysis' OR ENIGMA OR 'U54 EB020403' OR U54EB020403 OR 'Framingham Offspring Study' OR fos OR 'N01 HC25195' OR N01HC25195 OR 'Human Connectome Project' OR hcp OR U54MH091657 OR U01AG052564 OR 'U54 MH091657' OR 'U01 MH109589' OR 'U01 AG052564' OR 'imagen study' OR IMAGEN OR 'MRF_MRF 058 0009 RG DESR C0759' OR 'national consortium on alcohol and neurodevelopment in adolescence' OR NCANDA OR 'U24 AA021695' OR 'UK Biobank' OR MC_QA137853 OR MC_PC_17228’

Quality Assessment Items from the The NIH Quality Assessment Tool for Observational Cohort and Cross-Sectional Studies:

1. **Was the research question or objective in this paper clearly stated?**
2. **Was the study population clearly specified and defined?**
3. **Was the participation rate of eligible persons at least 50%?**
4. **Were all the subjects selected or recruited from the same or similar populations (including the same time period)? Were inclusion and exclusion criteria for being in the study prespecified and applied uniformly to all participants?**
5. **Was a sample size justification, power description, or variance and effect estimates provided?**
6. **For the analyses in this paper, were the exposure(s) of interest measured prior to the outcome(s) being measured?**
7. **Was the timeframe sufficient so that one could reasonably expect to see an association between exposure and outcome if it existed?**
8. **For exposures that can vary in amount or level, did the study examine different levels of the exposure as related to the outcome (e.g., categories of exposure, or exposure measured as continuous variable)?**
9. **Were the exposure measures (independent variables) clearly defined, valid, reliable, and implemented consistently across all study participants?**
10. **Was the exposure(s) assessed more than once over time?**
11. **Were the outcome measures (dependent variables) clearly defined, valid, reliable, and implemented consistently across all study participants?**
12. **Were the outcome assessors blinded to the exposure status of participants?**
13. **Was loss to follow-up after baseline 20% or less?**
14. **Were key potential confounding variables measured and adjusted statistically for their impact on the relationship between exposure(s) and outcome(s)?**

**Note: Response options for all questions were: Yes, No, Cannot Determine, Not Reported, N/A**
